# Evaluation and Management Principles for Chronic Right Heart Failure and Tricuspid Regurgitation

**DOI:** 10.1007/s11897-025-00720-1

**Published:** 2025-10-27

**Authors:** Revathy Sampath-Kumar, Andreas Rück, Aristomenis Manouras, Ori Ben-Yehuda, Lars H. Lund, Bahira Shahim

**Affiliations:** 1 Department of Cardiovascular Medicine, University of California, La Jolla, San Diego, CA USA; 2 Division of Cardiology, Department of Medicine, Karolinska Institutet, Solna, S1:02, 171 76, Stockholm, Sweden; 3 Heart and Vascular Theme, Karolinska University Hospital, Stockholm, Sweden

**Keywords:** Right heart failure, Tricuspid regurgitation, Management, Imaging, Intervention

## Abstract

**Purpose of the Review:**

This review provides an updated summary of the evaluation and management principles of chronic right heart failure (RHF) and tricuspid regurgitation (TR), with a focus on evolving diagnostic approaches and the role of transcatheter tricuspid valve interventions (TTVI).

**Recent Findings:**

Chronic RHF and TR frequently coexist and are associated with significant morbidity and mortality. Their interplay is both complex and bidirectional. TR leads to right ventricular (RV) volume overload, while RV remodeling in RHF promotes TR progression, primarily through annular dilatation and leaflet tethering. Advances in imaging modalities, including 3D echocardiography and cardiac magnetic resonance, have improved the evaluation of RV function and TR severity. Additionally, a refined TR grading system, now encompassing "massive" and "torrential" categories, enables more precise severity classification, which is particularly important for evaluating treatment response in device trials. Early identification of TR and RHF is crucial, and optimal management relies on understanding the underlying mechanisms, disease progression, and available treatment options. Although medical therapy for RHF and TR remains limited, TTVI offers an emerging alternative for selected patients. However, identifying appropriate candidates and the optimal timing for intervention remain key challenges.

**Summary:**

Timely diagnosis of RHF and TR, identification of the underlying causes, and comprehensive risk stratification, along with early referral to a multidisciplinary heart team, are critical for optimizing patient outcomes. Further research is needed to better define selection criteria and timing for TTVI.

## Introduction

Right heart failure (RHF) and tricuspid regurgitation (TR) frequently coexist and are often underdiagnosed, despite their strong associations with reduced quality of life, increased risk of heart failure hospitalization (HFH) and mortality [1–5]. The interplay between RHF and TR is both complex and bidirectional. TR contributes to RHF by imposing chronic volume overload on the right ventricle (RV), while RHF promotes TR through RV remodeling that induces structural and functional changes in the tricuspid valve (TV) [3, 6]. These changes, primarily annular dilatation and leaflet tethering, contribute to the vast majority of TR cases. Together, RHF and TR create a vicious cycle in which each condition exacerbates the other, accelerating disease progression, and posing significant diagnostic and therapeutic challenges.

**Fig. 1:**
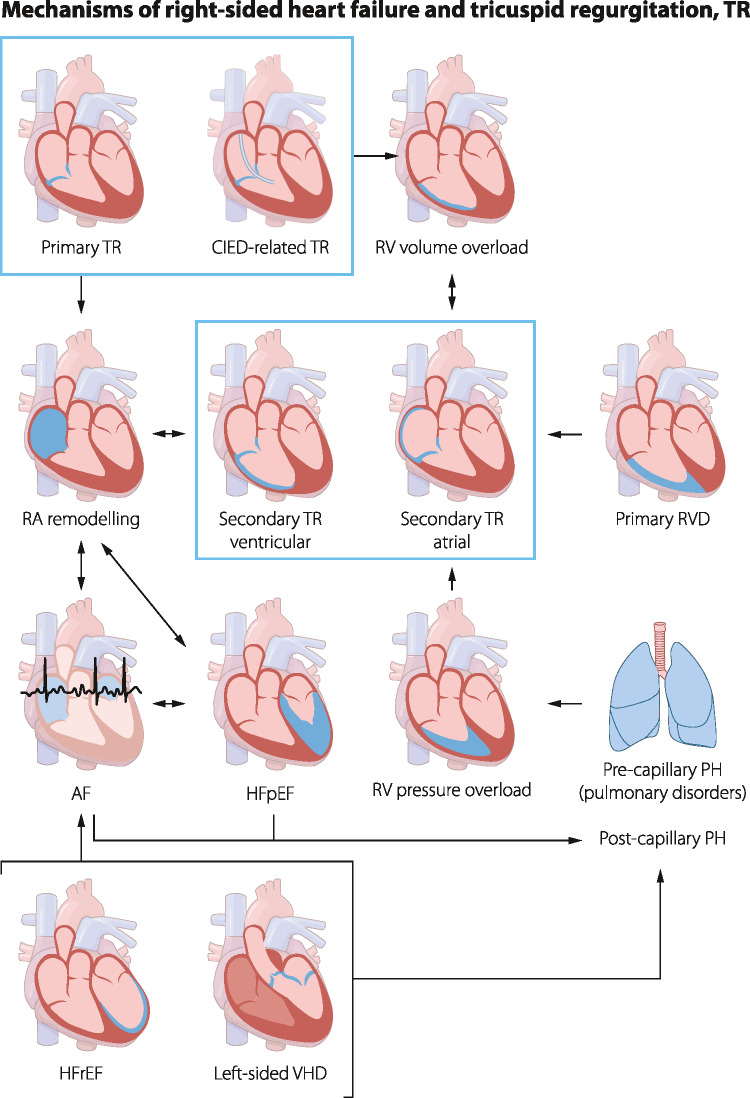
Mechanisms of right-sided heart failure and tricuspid regurgitation. TR = Tricuspid Regurgitation; RVD = Right Ventricular Dysfunction; CIED = Cardiac Implantable Electronic Device; HFpEF = HF with Preserved Ejection Fraction; PH = Pulmonary Hypertension; HFrEF = HF with Reduced Ejection Fraction; VHD = Valvular Heart Disease

The causes of RHF and TR are multifactorial (Fig. 1) including left ventricular dysfunction (regardless of ejection fraction), left-sided valvular heart disease, pulmonary hypertension, intrinsic RV pathology, and pulmonary disorders [1, 3, 7]. Right atrial involvement is also frequently observed, either as a consequence of RHF or as a contributing factor. In patients with long-standing atrial fibrillation or HF with preserved ejection fraction (HFpEF), right atrial remodeling, often accompanied by TR, can be observed and may further drive the progression of RHF [8, 9]. TR related to implantable electronic device (CIED) impingement is common [10]. Although less frequent, TR can also result from primary structural abnormalities of the TV.

Prevalence estimates for RHF and TR vary substantially, reflecting differences in definitions, diagnostic criteria, and study populations. In HF with reduced ejection fraction (HFrEF), RV dysfunction is reported in 19% to 77% of patients [11], whereas in HFpEF, estimates range from 18 to 28% [2]. TR is also widely prevalent particularly in older populations and those with atrial fibrillation. Moderate to severe TR affects 0.5% to 7% of the general population [12–15]. In an analysis of the ESC HF Long-Term Registry, isolated moderate to severe TR was found among 4% of patients with HFrEF and 10% with HFpEF [16]. When combined with mitral regurgitation, its prevalence was 12% in HFrEF and 8% in HFpEF. Importantly, significant TR is independently associated with worse clinical outcomes, regardless of RHF or pulmonary pressures, with a reported 5-year overall mortality rate of approximately 50% [17, 18]. However, a stepwise increase in mortality risk has been observed, with the poorest prognosis seen in those exhibiting RV and LV dysfunction and especially among those with pulmonary hypertension [19].

**Fig. 2:**
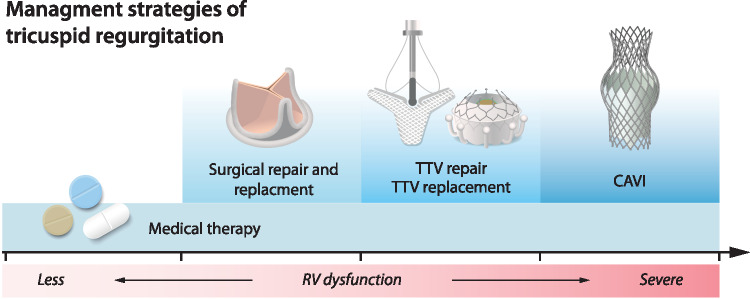
Management strategies of tricuspid regurgitation. TTV = Transcatheter Tricuspid Valve; CAVI = Caval Valve Implantation; RV = right ventricular

In clinical practice, RHF and TR are often diagnosed late, when symptoms are advanced and therapeutic options are limited (Fig. 2). At this point, surgical correction of TR carries high perioperative risk, and medical therapy only provides limited symptomatic relief. While transcatheter tricuspid valve interventions (TTVI) are emerging as promising alternatives, patient selection and determining the optimal timing and approach for these procedures remain significant clinical challenges.

This review aims to provide a comprehensive overview of the current evaluation and management principles for chronic RHF and TR, with a focus on evolving diagnostic approaches and interventional therapeutic strategies.

### Pathophysiology and Etiology of Chronic Right Heart Failure

Chronic RHF arises from primary RV dysfunction, which can manifest as an intrinsic impairment of contractility and/or lusitropy, or, more commonly, secondarily due to pressure or volume overload, often in the setting of left-sided HF (Table 1) [6]. Primary RV dysfunction can be attributed to etiologies such as myocardial infarction, myocarditis, cardiomyopathy, post-surgical states, or the presence of a left ventricular assist device. Secondary RV dysfunction resulting from pressure overload is caused by pre-capillary pulmonary hypertension, post- or combined pre-and post- capillary pulmonary hypertension (secondary to left HF), pulmonary embolism, various forms of lung disease, or pulmonary valve stenosis. Secondary RV dysfunction due to volume overload can result from tricuspid or pulmonic regurgitation, intracardiac left-to-right shunts, and extracardiac arteriovenous shunts. The degree of RV dysfunction is determined based on echocardiographic parameters, though cardiac MRI offers more precise quantification [20].

**Table 1:** Causes of chronic right sided heart failure

Decreased RV contractility	RV volume overload	RV pressure overload
RV cardiomyopathy	TR	Precapillary pulmonary hypertension
ARVC and other non-ischemic cardiomyopathies	Pulmonary regurgitation	Chronic thromboembolic pulmonary hypertension
Systemic sclerosis	Congestion from decompensated left heart failure	Pulmonary stenosis
Possibly obesity	Intracardiac left to right shunt	Left-sided valvular heart disease
	Extracardiac arteriovenous shunts	Restrictive cardiomyopathy
	Left-sided heart disease	
	Single ventricle	
Ebstein anomaly		

*RV* Right Ventricle, *ARVC* Arrhythmogenic Arrhythmogenic Right Ventricular Cardiomyopathy

In response to chronic stress, the RV undergoes both adaptive and maladaptive changes. Initially, the RV may exhibit hypertrophy and increased contractility. However, prolonged pressure or volume overload can lead to maladaptive remodeling, including RV dilatation and myocardial fibrosis, ultimately impairing both systolic and diastolic function [6, 21]. This RV dysfunction also heavily influences LV function due to the close interaction through the shared interventricular septum and pericardium. RV dilatation and dysfunction can shift the interventricular septum towards the LV, impairing LV filling and systolic function and thereby contributing to biventricular failure [6, 21].

RV dysfunction has significant systemic consequences, primarily due to “backward failure” and venous congestion. Elevated central venous pressure leads to multi-organ dysfunction, particularly affecting the liver and kidneys, and manifests clinically as peripheral edema. Refractory congestion is common among patients discharged from a HFH [22, 23], TR is among the stongest independent predictors of residual congestion [23], and isolated TR may be associated with higher NTproBNP and past HFH than isolated MR and even combined MR/TR [16].

### Pathophysiology and Etiology of Tricuspid Regurgitation

TR is categorized into primary, secondary, and CIED-induced etiologies [24, 25] (Table 2). Primary TR, accounting for a minority of cases (5–10%), results from intrinsic structural abnormalities of the TV apparatus. These include congenital anomalies such as Ebstein's anomaly, infective endocarditis, rheumatic heart disease, carcinoid heart disease, and myxomatous degeneration [3]. Secondary TR constitutes the vast majority of TR cases (80%) and is characterized by structurally normal valve leaflets that fail to coapt properly due to right atrial or RV dilatation.

**Table 2:** Causes of tricuspid regurgitation

Classification	Etiology
**Secondary (functional) tricuspid regurgitation (approximately 80% of cases)**	
Valve structures are anatomically normal and valve dysfunction is secondary to atrial or ventricular remodelling and dysfunction	
Ventricular secondary TR	• Postcapillary PH due to left ventricular disease or valvular disease • Precapillary PH due to primary pulmonary arterial or parenchymal disease (PAH, chronic lung disease or CTPEH) • Primary RV dysfunction or remodelling (due to myocardial infarction or RV cardiomyopathy)
Atrial secondary TR	• Right atrial or tricuspid annular dilation (HFpEF, atrial fibrillation, aging)
**Primary or mixed tricuspid regurgitation (5–10% of cases)**	
Valve structures are abnormal	
Primary TR	• Chordal elongation/rupture • Papillary muscle rupture (trauma) • Excessive leaflet motion (myxomatous disease) • Leaflet perforation (endocarditis) • Leaflet retraction (rheumatic, inflammatory diseases) • Leaflet infiltration
**CIED-related (approximately 10–15% of cases)**	
TR is caused by the interaction with intracavitary leads	
Lead-related TR	• Leaflet impingement, perforation, or valvular or subvalvular adhesions or restrictions, laceration (post-extraction) • Incidental (presence of CIED without interference in valvular apparatus)

*PH* Pulmonary hypertension, *PAH* = Pulmonary arterial hypertension, *CTPEH* Chronic thromboembolic pulmonary hypertension, *TR* tricuspid regurgitation, *HFpEF* HF with preserved ejection fraction, *CIED* cardiac implantable electronic device

Atrial secondary TR is predominantly a consequence of right atrial remodeling secondary to chronic atrial fibrillation. Ventricular secondary TR, which carries a less favorable prognosis independent of other clinical and echocardiographic factors [19], arises from RV dilatation and remodeling. The prevalence and clinical significance of secondary TR are particularly pronounced in the context of chronic RHF, where it frequently contributes to disease progression and adverse outcomes [19].

The mechanisms underlying ventricular secondary TR involve both tricuspid annular dilatation and leaflet tethering. Right ventricular dilatation leads to an increase in the tricuspid annular dimensions, disrupting the normal geometry of the valve orifice [3]. Additionally, as the RV dilates and becomes more spherical, the papillary muscles are displaced, causing tethering of the TV leaflets which impairs coaptation resulting in regurgitant flow.

CIED-associated TR occurs due to the physical presence right sided leads impinging on the TV leaflets or subvalvular apparatus, or as a consequence of pacing-induced RV dysfunction [26]. The growing utilization of CIEDs in an aging population is increasing the clinical relevance of CIED-associated TR [10].

The classification of TR severity has been expanded beyond the traditional mild, moderate, and severe grading to include massive and torrential categories [27]. This refined grading is particularly relevant in the context of TTVI clinical trials, where the reduction in TR grade serves as an important outcome measure. Baseline massive and torrential TR are associated with an increased risk of the composite outcome of mortality and HFH following TTVI, primarily driven by the latter [28]. However, procedural success rates in these patients are similar to those with severe TR, and procedural success leads to improved clinical outcomes irrespective of TR grade. It is crucial to note, however, that eligibility for TTVI is dependent on favorable anatomical characteristics. In a significant number of cases with massive and torrential TR, large coaptation gaps render these patients ineligible for TTVI, thereby limiting therapeutic options apart from surgery [29].

TR and RV dysfunction have a complex, bidirectional relationship. RV dysfunction can induce RV and tricuspid annular dilatation, leading to or worsening secondary TR. The resulting increase in RV preload due to the regurgitant volume further exacerbates RV dilatation and impairs RV function, creating a detrimental positive feedback loop that accelerates the progression of HF and valvular dysfunction. While both conditions drive one another, RHF is more commonly the cause of TR than TR is the primary cause of RHF. In patients with TR, more advanced stages of RHF are independently associated with increased long-term mortality [19, 30], and the degree of TR often correlates directly with the extent of RV dilatation [13]. Early identification and implementation of appropriate treatment strategies are therefore essential to interrupt this vicious cycle and improve patient outcomes.

### Evaluation of Chronic Right Heart Failure and Tricuspid Regurgitation

#### Clinical Assessment

The clinical evaluation of patients with suspected RHF and TR necessitates a detailed history and thorough physical examination. The initial symptoms of RHF and TR can be non-specific and insidious, frequently leading to delays in establishing a diagnosis and initiating appropriate treatment [24]. Notably, even in the presence of severe TR during the earlier stages, patients may present with subclinical findings or report symptoms such as fatigue, exercise intolerance, and dyspnea, which may be misattributed to other underlying conditions. As the disease progresses, systemic fluid retention results in progressive end-organ damage secondary to venous congestion, reduced cardiac output, and inadequate tissue perfusion. At this stage, patients typically exhibit more overt clinical manifestations [3].

A comprehensive physical examination should focus on identifying key clinical signs indicative of elevated central venous pressure, including jugular venous distension, as well as the presence and extent of peripheral edema, ascites, and the characteristic low-amplitude holosystolic murmur associated with TR. The New York Heart Association functional classification should be utilized to categorize functional capacity, as this provides valuable prognostic information in the context of RHF [30].

#### Diagnostic Modalities

Transthoracic echocardiography (TTE) serves as the primary imaging modality for the initial evaluation of the right heart and TV (Table 3). Quantitative echocardiographic parameters for assessing RV function include tricuspid annular plane systolic excursion, tissue doppler imaging-derived systolic velocity at the lateral tricuspid annulus, RV fractional area change (RVFAC), and RV free wall strain [20]. The presence and severity of TR can be assessed on TTE using color doppler, doppler effective regurgitant orifice area, and vena contracta. TR severity should be assessed using a multi-parametric and semi-quantitative or quantitative approach as a purely qualitative approach can lead to underdiagnosis [31]. Due to the complex anatomy and location of the RV, visualization can be challenging.

**Table 3:** Imaging modalities for diagnosis of right-sided HF and tricuspid regurgitation and for guidance of tricuspid interventions

Imaging modality	Applications
Transthoracic echocardiography	• Grading of TR severity • Assessment of tricuspid valve pathology • Evaluation of right ventricular function • Diagnosis of pulmonary hypertension • Pacemaker/defibrillator lead location and evaluation of tricuspid valve leaflet lead impingement
Transesophageal echocardiography	• Exclusion of intracardiac thrombus/masses • Pacemaker/defibrillator lead location and evaluation of tricuspid valve leaflet lead impingement • Procedural guidance
Intracardiac echocardiography	• Procedural guidance when insufficient TEE quality or contraindications to esophageal intubation
Cardiac computed tomography	• Assessment of annular shape, dimensions and annular calcification • Assessment of the relationships of the tricuspid annulus to surrounding structures (particularly the RCA) • Definition of optimal procedural fluoroscopic angulations • Evaluation of specific annular anchor points in relation to tricuspid leaflet hinge points and coronary arteries • Evaluation of RCA status • Evaluation of the relationship between IVC and tricuspid valve annulus • Determination of the location of pacemaker/defibrillator leads
Coronary angiography/fluoroscopy	• Evaluation of RCA status • Navigation and control of patency of the RCA if a device is anchored to the tricuspid annulus • Orientation and device placement/deployment
Cardiac magnetic resonance imaging	• Grading of TR severity • Evaluation of RV function • Assessment of myocardial fibrosis
Right heart catheterization	• Assessment of presence and category of pulmonary hypertension • Evaluation of right heart function

*TR* tricuspid regurgitation, *IVC* inferior vena cava, *RCA* right coronary artery, *RV* right ventricle

Transesophageal echocardiography (TEE) offers superior visualization of the TV apparatus, facilitating the determination of TR mechanisms, leaflet morphology, and coaptation. TEE is particularly valuable for assessing anatomical suitability for TTVI and for guiding transcatheter procedures.

Three-dimensional echocardiography provides an en face view of the TV annulus and leaflets, enabling more accurate quantification of annular size and shape, as well as the extent of leaflet tethering compared to two-dimensional echocardiography. Three-dimensional echo data can be rotated and sliced to assess device leads and TV leaflets in multiple planes which can assist with determining the etiology of TR. Furthermore, three-dimensional echocardiography allows for accurate and reproducible quantification of RV volume and ejection fraction, with less variability than two-dimensional echocardiography and comparable accuracy to cardiac MRI [32].

Cardiac MRI is the reference standard for assessment of RV size, RV ejection fraction, and RV mass [33]. Additionally, MRI enables tissue characterization to identify underlying myocardial abnormalities. Four-dimensional flow MRI offers non-invasive quantification of blood flow velocities across the TV. MRI is particularly advantageous when echocardiographic image quality is suboptimal or a detailed evaluation of RV morphology, function, and hemodynamics is required due to its superior spatial resolution and reduced susceptibility to acoustic window limitations.

Invasive hemodynamic assessment via right heart catheterization (RHC) permits direct measurement of intracardiac and pulmonary pressures, including the pulmonary vascular resistance and pulmonary capillary wedge pressure, which cannot be measured non-invasively. RHC should be performed after optimizing volume status, as intravascular volume significantly influences TR severity and the degree of RHF. RHC is essential to exclude significant pre-capillary pulmonary hypertension, as echocardiographic assessment of pulmonary artery pressures can be discordant with invasive measurements in severe TR due to the presence of rapid pressure equalization between the RV and right atrium [34]. Current practice guidelines therefore recommend invasive RHC for accurate pulmonary artery pressure and pulmonary vascular resistance measurement and pulmonary hypertension diagnosis [35]. The presence of pre-capillary pulmonary hypertension may contraindicate or necessitate careful consideration before TV interventions. Pre-capillary pulmonary hypertension has been associated with a higher mortality risk following TTVI compared to post-capillary pulmonary hypertension [36]. Increased mean pulmonary artery pressure, transpulmonary gradient, pulmonary vascular resistance, and RV stroke work index are also hemodynamic predictors of mortality following TTVI [36].

The pulmonary artery pulsatility index (PAPi), calculated as the ratio of pulmonary artery pulse pressure to right atrial pressure, is indicative of RV function, with lower PAPi correlating with worse survival in patients with TR [37]. Right ventricular to pulmonary artery (RV-PA) coupling, which assesses RV systolic function in relation to pulmonary artery pressure, is also a prognostic marker in patients with severe TR undergoing TTVI [38]. While PAPi and RV-PA coupling can be estimated non-invasively by echocardiography, invasive assessment via RHC provides greater accuracy. This underscores the value of invasive hemodynamic evaluation in specific clinical scenarios for a comprehensive understanding of hemodynamics and volume status, particularly in pre-interventional planning.

### Medical Management of Chronic Right Heart Failure and Tricuspid Regurgitation

The medical management of TR is limited to the use of diuretics to address fluid overload and the treatment of any underlying conditions contributing to the TR or RHF. Loop diuretics are a Class I guideline recommendation for the optimization of fluid status [39], and careful monitoring of volume status is essential to minimize the risk of HF-related hospitalizations. There are no pharmacologic therapies that have been proven to improve clinical outcomes in patients specifically with chronic RHF and TR. However, in individuals who have concomitant left ventricular dysfunction, adherence to guideline-directed medical therapy (GDMT) for HF has been associated with a reduction in the severity of TR [40]. For patients with coexisting atrial fibrillation, the restoration and maintenance of sinus rhythm has been observed to correlate with a decrease in the grade of TR [41]. Targeted medical management of underlying pulmonary hypertension has also demonstrated the potential to induce reverse remodeling of the RV and improve the degree of TR [42]. In CIED-mediated TR, lead extraction is a potential intervention, although its benefit is limited when the TR is not primarily lead-related or in the presence of leaflet damage [26].

### Interventional Therapies for Tricuspid Regurgitation

#### Surgical Tricuspid Valve Repair or Replacement

Surgical treatment approaches for TR include annuloplasty ring repair and valve replacement depending upon anatomic suitability and surgical expertise. For primary TR, guidelines give a Class I recommendation for surgery among patients with severe TR and a Class IIa recommendation for surgery among patients with moderate TR undergoing left-sided valve surgery [43]. There is also a Class I recommendation for surgery in symptomatic patients with isolated severe primary TR without severe RV dysfunction. For secondary TR, guidelines give a Class I recommendation for surgery among patients with severe TR undergoing left-sided valve surgery. There is a Class IIa recommendation for isolated TR surgery in patients with severe secondary TR who are symptomatic or have RV dilatation without severe RV or LV dysfunction or severe pulmonary hypertension.

There is a higher mortality risk associated with surgical treatment of tricuspid valve disease compared to aortic and mitral valve interventions [44]. Isolated tricuspid valve surgery is rarely performed and is associated with an in-hospital mortality of 8–10%, higher with replacement than repair [45–47]. The risk of mortality is higher later in the disease course and among older patients and those with RV dysfunction, highlighting the importance of prompt diagnosis and referral [47]. Historically, poor outcomes from TR surgery were attributed to pressure overload on an already severely compromised RV. However, this view is shifting with the emergence of percutaneous interventions.

#### Transcatheter Tricuspid Valve Interventions

Transcatheter therapies have emerged as potential treatment options for TR, particularly in patients deemed to be at prohibitive or high surgical risk [48, 49]. Current guidelines provide a Class IIb recommendation to consider transcatheter treatment for inoperable patients at specialized heart valve centers with expertise in tricuspid valve disease [43]. Transcatheter approaches encompass leaflet approximation, direct annuloplasty, and transcatheter valve replacement, with the selection based on individual anatomical suitability (Table 4).

**Table 4:** Criteria for treatment strategies and device selection in tricuspid regurgitation

Device system	Favorable/feasible anatomy	Constraints of the technologies
T-TEER	• Moderate annular dilatation • Septolateral coaptation gap ≤ 8.5 mm, coaptation depth > 10 mm • Anteroseptal or posteroseptal jet location	• Coaptation gap • Leaflet thickening/shortening/perforation • Dense chordae with marked leaflet tethering • Poor echocardiographic leaflet visualization • CIED RV lead leaflet impingement
TTVA	• Large annulus (annular dilatation as primary mechanism of TR) • Septolateral coaptation gap > 8.5 mm, coaptation depth < 10 mm • Central jet location • Sufficient landing zone for anchoring	• Annular proximity of RCA • Severe tethering • Poor echocardiographic annular visualization • Device complexity • CIED RV lead leaflet impingement
TTVR	• Large annulus • Septolateral coaptation gap > 8.5 mm, coaptation depth > 10 mm	• Annulus size and shape • Right ventricular dimensions • Subvalvular apparatus • Inferior vena cava angle
Orthotopic valve implantation	• Previous surgical repair or bioprosthetic valve replacement • Leaflet thickening/shortening • CIED RV lead (with or without leaflet impingement) • Any leaflet morphology	• Excessive annular dilation (exceeding device size) • Unfavorable device angle of approach • Severe RV dysfunction
Heterotopic valve implantation	• Appropriate caval diameters • No option for direct valve treatment	• Proximity of the right atrium to the orifice of the hepatic veins (< 10–12 mm) • Severely increased pulmonary artery and right atrial pressures (risk of fracture of bicaval valved stents)

*T-TEER* tricuspid transcatheter edge-to-edge repair, *TTVA* transcatheter tricuspid annuloplasty, *TTVR* transcatheter tricuspid valve replacement, *CIED* cardiac implantable electronic device, *RV* right ventricle

Tricuspid transcatheter edge-to-edge repair (TEER) is the most widely adopted technique due to its relative safety, availability, and procedural ease. The prospective randomized controlled TRILUMINATE (The Trial to Evaluate Cardiovascular Outcomes in Patients Treated with the Tricuspid Valve Repair System) trial demonstrated the safety of TEER and its efficacy in reducing TR severity. While TEER was associated with improvements in patient quality of life, no significant benefit in mortality or HFH was observed [50].

More recently, the TRISCEND II (Edwards EVOQUE Transcatheter Tricuspid Valve Replacement: Pivotal Clinical Investigation of Safety and Clinical Efficacy using a Novel Device) trial, a prospective randomized controlled trial evaluating transcatheter tricuspid valve replacement using the EVOQUE valve compared to medical therapy, demonstrated superiority of valve replacement for the hierarchical primary outcome. This benefit was driven by improvements in patient symptoms and quality of life, with greater improvements noted in patients with massive or torrential TR, better RV function, and higher functional capacity [51].

The Cardioband device is the only currently approved direct annuloplasty system. The TriBAND (Tricuspid Cardioband) study demonstrated sustained TR reduction and improvements in quality of life measures [52]. However, early experience with the device was associated with a notable rate (up to 15%) of right coronary artery perforation and occlusion, highlighting the importance of operator experience.

Given the complexity of these procedures, the potential for complications, and the remaining uncertainty regarding their impact on “hard” outcomes, TTVI necessitates execution at experienced centers with comprehensive support from interventional cardiology, cardiothoracic surgery, advanced HF specialists, and advanced cardiac imaging.

While propensity-matched cohort studies have suggested a potential association between TTVI and improved survival, as well as a lower incidence of HFH compared to medical therapy alone [53], randomized controlled trials have yet to demonstrate a benefit beyond functional status and quality of life. Some studies have indicated RV reverse remodeling following TTVI [38, 53]. Appropriate patient selection, considering the complete clinical context including age, comorbidities, and patient preferences, is paramount, emphasizing the importance of shared decision-making and a multidisciplinary heart team approach. The identification of patients who will derive the greatest benefit from these catheter-based procedures and the optimal timing for intervention remain areas of active investigation [49]. Notably, a mortality benefit with TTVI has been reported in patients with HF with mildly reduced ejection fraction but not in those with more severe left ventricular dysfunction [54]. Ongoing research continues to explore the long-term efficacy, optimal patient selection criteria, and the role of novel devices in this rapidly evolving field.

### Future Directions

Several key areas warrant further investigation to advance the evaluation and management of chronic RHF and TR. The investigation of medical therapies targeting RHF is crucial, given the current lack of proven pharmacologic interventions for this condition. Exploring novel therapeutic targets at the molecular and cellular levels may lead to the development of targeted therapies aimed at preventing disease progression and improving outcomes.

Optimizing patient selection criteria and improving outcomes for TTVI represent another area for future research. The analysis of data from the international TriValve registry, which tracks procedural outcomes with TTVI, has been instrumental in characterizing factors associated with procedural success and assessing long-term outcomes [55]. Prospective randomized controlled trials with long-term follow-up are needed to definitively establish the role of TTVI in improving survival and reducing HFH beyond patient reported outcomes. Advancements in device technology, enhanced procedural imaging guidance with 3D intracardiac echocardiography, and accumulated operator experience are expected to improve TTVI outcomes in the coming years.

Improving risk stratification and prognostic models for patients with RHF and TR is also essential. The application of artificial intelligence (AI)-enabled deep learning workflows has shown promise in detecting significant TR at scale, potentially aiding in earlier identification, particularly in underserved communities and by non-cardiology providers [56]. Additionally, AI-enabled assessment of RV-PA coupling from echocardiographic data holds potential for refining risk stratification in patients undergoing TTVR [57].

## Conclusion

Chronic RHF and TR exhibit a significant interplay. Timely identification through TTE and subsequent referral to a comprehensive heart team are of paramount importance. Establishing and treating the underlying cause is crucial, considering the common coexistence of these entities with left-sided HF, left-sided valvular disease, atrial fibrillation, and pulmonary hypertension. Medical management centers on the optimization of volume status. Surgical TV repair or replacement is warranted in the presence of concomitant left-sided valvular disease. TTVI offers a potential avenue for patients with high surgical risk, demonstrating efficacy in decreasing TR grade and enhancing quality of life. However, ongoing challenges exist regarding the optimal selection of patients for interventions and the improvement of early diagnosis in community settings, especially within underserved populations. Ultimately management strategies must be tailored to the individual patient, considering their specific symptom profile and therapeutic objectives within a framework of shared decision-making.

## Key References

- Adamo M, Chioncel O, Benson L, Shahim B, Crespo-Leiro MG, Anker SD, et al. Prevalence, clinical characteristics and outcomes of heart failure patients with or without isolated or combined mitral and tricuspid regurgitation: An analysis from the ESC-HFA Heart Failure Long-Term Registry. Eur J Heart Fail. 2023 Jul;25(7):1061–1071.
  - This is the largest study to date to assess the prevalence, predictors and prognostic implications of tricuspid regurgitation across the spectrum of heart failure categories.
- Wang Wang N, Fulcher J, Abeysuriya N, McGrady M, Wilcox I, Celermajer D, Lal S. Tricuspid regurgitation is associated with increased mortality independent of pulmonary pressures and right heart failure: a systematic review and meta-analysis. Eur Heart J. 2019 Feb 1;40(5):476–484.
  - This study demonstrates that moderate or severe tricuspid regurgitation is associated with an increased mortality risk, independent of pulmonary pressures and right ventricular dysfunction.
- Sanz J, Sánchez-Quintana D, Bossone E, Bogaard HJ, Naeije R. Anatomy, Function, and Dysfunction of the Right Ventricle: JACC State-of-the-Art Review. J Am Coll Cardiol. 2019 Apr 2;73(12):1463–1482.
  - The study summarizes current knowledge of right ventricular anatomic, structural, metabolic, functional, and hemodynamic characteristics in both the healthy heart as well as in right-sided and left-sided heart failure.
- Surkova E, Cosyns B, Gerber B, Gimelli A, La Gerche A, Ajmone Marsan N. The dysfunctional right ventricle: the importance of multi-modality imaging. Eur Heart J Cardiovasc Imaging. 2022 Jun 21;23(7):885–897.
  - The authors highlight the importance of using different imaging modalities in a complementary fashion to assess the right ventricular function.
- Sorajja P, Whisenant B, Hamid N, Naik H, Makkar R, et al. Transcatheter Repair for Patients with Tricuspid Regurgitation. N Engl J Med. 2023 May 18;388(20):1833–1842.
  - The TRILUMINATE Pivotal study showing that percutaneous tricuspid transcatheter edge-to-edge repair was safe for patients with severe tricuspid regurgitation, reduced the severity of tricuspid regurgitation, and was associated with an improvement in quality of life.
- Hahn RT, Makkar R, Thourani VH, Makar M, Sharma RP, Haeffele C, et al. TRISCEND II Trial Investigators. Transcatheter Valve Replacement in Severe Tricuspid Regurgitation. N Engl J Med. 2025 Jan 9;392(2):115–126.
  - The TRISCEND II study showing that for patients with severe tricuspid regurgitation, transcatheter tricuspid-valve replacement was superior to medical therapy alone for a hierarchical composite primary outcome, driven primarily by improvements in symptoms and quality of life.

## Data Availability

No datasets were generated or analysed during the current study.

## Abbreviations

TR: Tricuspid regurgitation
RHF: Right HF
TTVI: Transcatheter tricuspid valve interventions
RV: Right ventricle
TV: Tricuspid valve
CIED: Implantable electronic device
TTE: Transthoracic echocardiography
TEE: Transesophageal echocardiography
MRI: Magnetic resonance imaging
RHC: Right heart catheterization
PAPi: Pulmonary artery pulsatility index
RV-PA: Right ventricular to pulmonary artery
TEER: Tricuspid transcatheter edge-to-edge repair
HFH: Heart failure hospitalization
HFpEF: HF with preserved ejection fraction
HFrEF: HF with reduced ejection fraction
